# Effectiveness of Virtual Reality in the Rehabilitation of Motor Function of Patients With Subacute Stroke: A Meta-Analysis

**DOI:** 10.3389/fneur.2021.639535

**Published:** 2021-05-05

**Authors:** Quan-cheng Peng, Ling Yin, Yi Cao

**Affiliations:** ^1^Department of Rehabilitation Medicine, Hanchuan People's Hospital, Hanchuan, China; ^2^Department of Pharmacy, Hanchuan People's Hospital, Hanchuan, China

**Keywords:** subacute stroke, rehabilitation, virtual reality, conventional therapy 3, meta

## Abstract

Stroke is a major cause of death and disability in adults. Conventional therapy (CT) has limited effectiveness, and therefore, various virtual reality (VR) rehabilitation programs have been designed. However, their efficacy in regaining motor function in patients with subacute stroke is questionable. Therefore, we conducted this meta-analysis to determine the efficacy of VR, compared to CT, in restoring motor function in this patient population. Up to October 10, 2020, nine electronic databases were searched for relevant articles reporting the effectiveness of VR in regaining motor function in patients with subacute stroke. This search was updated on March 7, 2021, with no additional added articles. The control group included CT, physical therapy, occupational therapy, or a combination of them. Effectiveness is defined as the positive change from baseline values to the last follow-up point. The Cochrane's revised risk-of-bias tool was used to determine the quality of included trials. A metaregression analysis was conducted to determine the effect of “time since last stroke” on reported outcomes. Publication bias and sensitivity analyses were also carried out. A total of 19 studies (17 randomized controlled trials, 1 cohort study, and 1 crossover trial) were included in the qualitative analysis, whereas 16 trials were meta-analyzed. A great improvement in motor function was noted in the VR group, when compared to preintervention values [standardized mean difference (SMD) = 1.14; 95% confidence interval (CI) = 0.77–1.52; *I*^2^ = 82%; *P* < 0.001]. When compared to CT, VR resulted in mild improvement in motor function (SMD = 0.47; 95% CI = 0.22–0.72; *I*^2^ = 75%; *P* < 0.001). However, upon trim-and-fill adjustment, this finding was deemed insignificant (SMD = 0.08; 95% CI = −0.16 to 0.33; *I*^2^ = 82.6%; *P* < 0.001). Ten studies had low risk, five had some concerns, three had high risk, and one had a moderate risk of bias. VR programs can be used jointly with CT for the rehabilitation of the motor function of patients with subacute stroke. However, more studies are still warranted to determine the effectiveness of these interventions in retaining the cognitive function and physical performance of such patients.

## Introduction

Stroke is a major cause of mortality and acquired disabilities in the adult population (1, 2). Recently, the risk of stroke-related mortality has been greatly reduced because of the improved accessibility to healthcare and stroke management protocols, which include recanalization therapy (3), decompression therapy (4), and stroke unit management (5). However, there is a remarkable increase in the number of neurologically impaired patients with significant disabilities (6). Of whom, only a few may regain some functionality in affected upper limb (UL) or lower limb (LL) (7, 8). This, in turn, will greatly impact the affected individuals' ability to self-care and their engagement in social activities. As most activities of daily living (ADLs) involve the use of ULs and LLs, it is of great importance to improve their functional state in post stroke patients.

In clinical practice, post stroke rehabilitation currently depends mainly on promoting neuroplasticity after brain injury (9, 10). In an attempt to maximize the effect of neuroplasticity, training must be based on learning repetitive, challenging, and motivating, as well as intense tasks (11, 12). In this context, conventional therapies (CTs), in the form of occupational or physical therapy, are commonly used to improve the motor function of affected limbs following brain injury (13–15). However, researchers have been studying other treatment options because conventional rehabilitation programs are often time-consuming and resource-intensive, and their outcomes rely mainly on the ability and prior training of the interventionist. Therefore, virtual reality (VR) has gained attention in the past decades for its potential benefits in promoting motor recovery in stroke survivors. Moreover, it has been reported that repetition, intensity, and dose in CT settings are not sufficient to reach plasticity-based optimal motor recovery (16). The aforementioned limitations drove the introduction of new options of potential benefit in regaining the motor function in affected individuals, such as VR.

VR-based interventions are now used as therapeutic options for promoting neurorehabilitation in patients with stroke, enabling patients to perform their daily activities, which are difficult to be carried out in a rehabilitative facility. Also, compared to the standard CT, VR therapy is designed to offer entertainment and joy, and therefore, it encourages patients to participate more in the rehabilitative program (17, 18). Moreover, VR programs can be carried out in clinical settings and at a low cost, and therefore, many patients can comply with them.

VR training therapy has been increasingly used in order to facilitate motor recovery in stroke survivors, and the majority of published articles investigated patients in the chronic stage. They also have shown that the use of VR is beneficial in this regard. For example, a recent systematic review and meta-analysis concluded that VR therapy could moderately improve the motor function of both ULs and LLs in patients with chronic stroke in comparison with CTs (19). Moreover, compared to CT, VR has been shown to result in a positive impact on balance as well (20).

That being said, the number of studies investigating the effect of VR-based interventions in patients with subacute stroke remains scarce (9, 21–23), and no conclusions have been reached in this regard. This topic is of great interest because (1) the plasticity of the brain remains good during the acute and subacute phase (24, 25) and (2) the effectiveness of rehabilitation therapy–induced neuroplasticity is limited in chronic stroke patients, particularly those who missed the window of opportunity that is present during the subacute phase (when the brain plasticity peaks) (26).

Therefore, we conducted the current investigation to systematically review the available high-quality evidence in the literature regarding the use of VR rehabilitation interventions, compared to CTs, in regaining the functional state of affected limbs among patients with subacute stroke.

## Materials and Methods

### Search Strategy and Study Selection

The study process was conducted following the accepted methodology recommendations of the PRISMA checklist for systematic review and meta-analysis where registration of the protocol is not mandated (27). An electronic database search was conducted for relevant studies published from inception until October 10, 2020, in nine databases: PubMed, Google Scholar, Scopus, Web of Science, The New York Academy of Medicine, Virtual health library, the System for Information on Gray Literature in Europe, ClinicalTrials.gov, and meta Register of Controlled Trials. Of note, this search was updated on March 7, 2021, to include any recently published relevant articles. However, no additional articles were found eligible.

For the purposes of conducting this research, we used the definition of the subacute phase of stroke as the period from 7 days to 6 months following the occurrence of stroke, as highlighted in the recent consensus of the Stroke Recovery and Rehabilitation Roundtable Taskforce (28). The search was conducted using the following keywords: (subacute stroke OR stroke) AND (virtual reality) AND (trial OR RCT or random OR randomized) and/or medical subject (MeSH) terms, as appropriate. We further did a manual search of references in our included articles to avoid missing relevant studies (29, 30). The search process was done based on the PICO framework: participants were any patient with subacute stroke (as defined above) affecting the motor function of upper or lower extremities, the interventions were VR-based therapies, the comparison was CTs, and treatment effectiveness was the outcome of interest. “Conventional therapy” could consist of usual CT, physical therapy, occupational therapy or a combination of any of them. “Effectiveness” was defined as the positive change from baseline values to the last follow-up point. The change in motor function was our primary outcome of interest; in the case of multiple scales, effectiveness was measured by the change in the scale that was reported as the primary outcome or most relevant to other studies (to maintain homogeneity). We also aimed to highlight the feasibility of using different VR programs in patients with subacute stroke.

We included all original studies that assessed the effectiveness of different VR programs in subacute stroke patients. However, articles were excluded according to the following exclusion criteria: (1) nonoriginal studies or nonhuman (*in vitro* or animal) studies; (2) duplicate records, overlapped data, or when data could not be reliably extracted; (3) incomplete reports; and (4) abstract-only articles, reviews, thesis, books, conference papers, or articles without available full texts.

The title and abstract screening were performed by four independent reviewers. Then, three independent reviewers performed a full-text screening to ensure the inclusion of relevant articles in our systematic review. Any disagreement was resolved by discussion and referring to the senior author when necessary.

### Data Extraction

Two authors developed the data extraction sheet using the Microsoft Excel software. Data extraction was performed by three independent reviewers using the Excel sheet. The fourth independent reviewer performed data checking to ensure the accuracy of extracted data. All disagreements and discrepancies were resolved by discussion and consultation with the senior author when necessary.

### Risk of Bias

Three independent reviewers evaluated the risk of bias of included studies. For randomized controlled trials, we decided to use Cochrane's revised quality assessment tool (ROB-II) (31). For non-randomized studies, the risk of bias in non-randomized studies of interventions (ROBINS-I) was used to assess the quality of included studies (32). Any discrepancy between the reviewers was solved by discussion.

### Statistical Analysis

All data were analyzed using the R software, version 4.0.2 (33). The standardized mean difference (SMD) effect size and its variance were calculated using the preintervention and postintervention data for both intervention and control groups (34, 35). Using a “meta” package, changes from baseline for both intervention and control were analyzed to calculate the pooled SMD and the corresponding standard errors (36). The SMD was used because of the difference in measurement methodology among included studies, and it is more generalizable than the mean difference (37).

The related 95% confidence intervals (CIs) of the computed effect size were calculated using a fixed- or random-effects model based on the extent of heterogeneity. To assess heterogeneity, Q statistics and *I*^2^ test were used, with *I*^2^ value higher than 50% or *P* < 0.05 considered significant (38). Moreover, we conducted Egger regression test to assess publication bias, which was considered significant when *P* < 0.10 (39, 40). Whenever publication bias was found, the trim-and-fill method of Duvall and Tweedie was applied to add studies that appeared to be missing (41) to enhance the symmetry. Furthermore, metaregression was used to explore the effect of “time passed since the last stroke” on the VR effectiveness (42). In the case of statistically significant results, a leave-one-out sensitivity analysis was performed by iteratively removing one study at a time to confirm that our findings were not driven by any single study (43).

## Results

### Search Results

**Figure 1:**
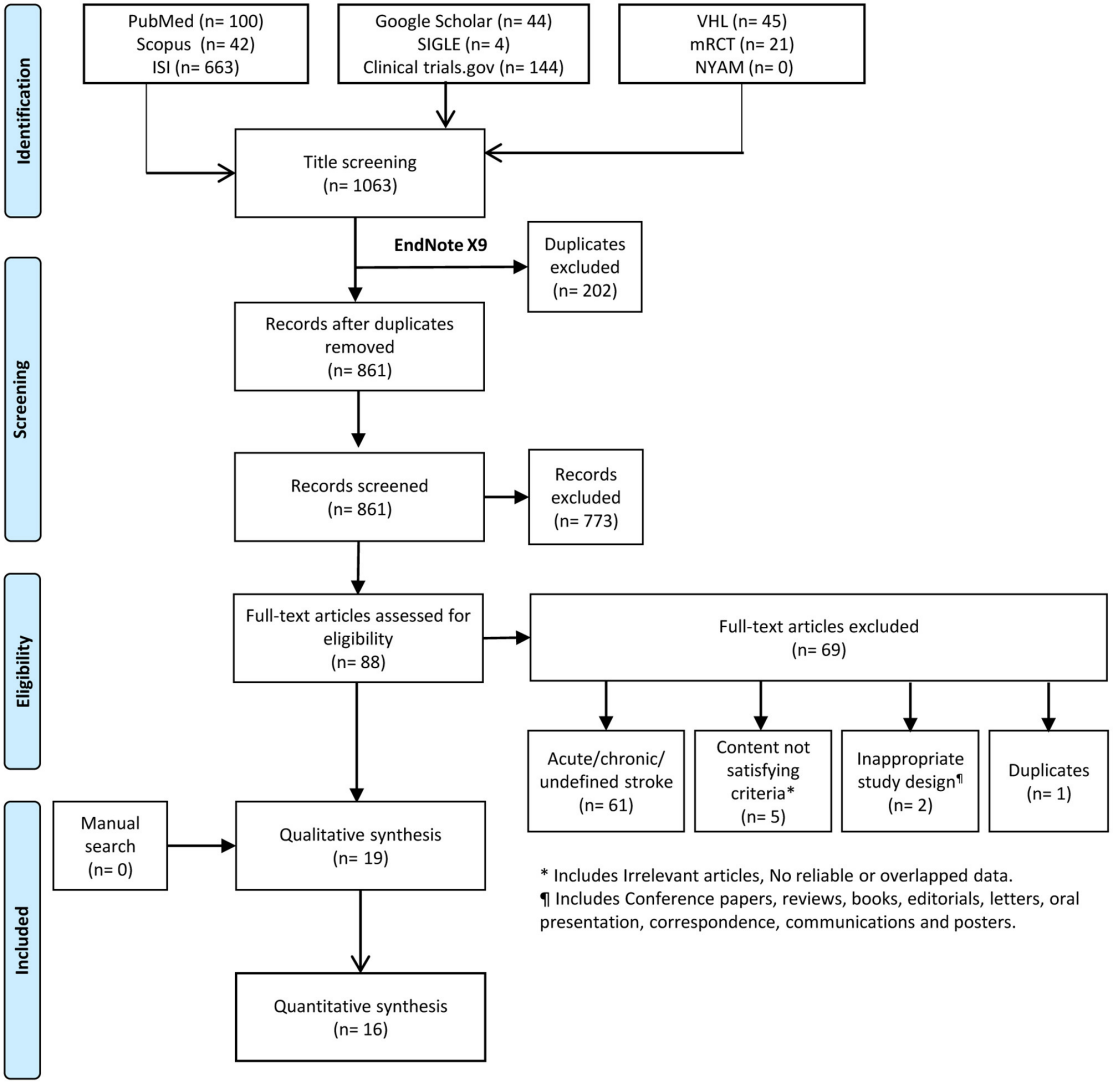
PRISMA flow diagram showing the process of the review.

We identified 861 records after excluding 202 duplicates using the Endnote X9 software. Title and abstract screening resulted in 88 records for further full-text screening. No articles were added after performing a manual search. Finally, a total of 19 studies were included in the systematic review, and 16 articles were eligible for meta-analysis (Figure 1).

### Study Characteristics

Nineteen studies compared VR vs. controls. According to the countries in which the included trials were conducted, four studies were conducted in Turkey, four in Korea, two in Norway, two in Italy, two in Australia, two in Canada, two in China, and one in Germany. Based on the design of included studies, 17 articles were randomized controlled trials, one was a cohort study, and the last one had a crossover design (Table 1).

**Table 1:** Baseline characteristics of included studies assessing VR in subacute stroke patients.

**Author, year**	**Country**	**Design**	**Sample Size**	**Male %**	**Age mean (SD) -inter- vention**	**Age (years): mean (SD) - comparison**	**Extremity**	**Assessment scales**	**Comparison group**	**Aim**	**Conclusion**
Mekbib, 2020 (44)	China	Cohort	21	NA	57.13 (± 4.45)	55 (± 7.85)	UE	Resting-state fMRI and FMA for UL	Healthy controls	To test the impacts of VR-based limb mirroring therapy (VRLMT) on brain reorganization and UE recovery in stroke patients with moderate to severe UE impairments.	Unilateral and bilateral limb mirroring exercise in an immersive virtual environment may enhance cortical reorganization and lead to improved motor function
Afsar, 2018 (45)	Turkey	RCT	35	57.14	69.42 (± 8.55)	63.44 (± 15.73)	UE	BBT, FMA for UE, B-stage, and FIM self-care score	Received 60 minutes of conventional therapy for upper extremity, 5 times per-week for 4 weeks	To evaluate the effect of the Microsoft Xbox360 Kinect video game system on UE motor functions for subacute stroke patients.	We found evidence that kinect-based game system in addition to conventional therapy may have supplemental benefit for stroke patients.
Kim, 2018 (22)	Korea	RCT	23	73.91	56.7 (± 17.8)	57.2 (± 15.0)	UE	BBT, B-stage, FMA, Korean version of modified BI, and TAC	Participated in a daily 30-minute occupational therapy session targeting the hemiparetic UE recovery based on the adaptive task practice (shaping) for 10 consecutive weekdays (5 days per week).	To prove the efficacy of the low-cost Kinect-based virtual rehabilitation (VR) system for UE recovery among patients with subacute stroke	Low-cost Kinect-based UE rehabilitation system was not more efficacious compared with sham VR. However, the compliance in VR was good and VR system induced more arm motion than control and similar activity compared with the conventional therapy, which suggests its utility as an adjuvant additional therapy during inpatient stroke rehabilitation.
Lee, 2016 (21)	Korea	RCT	10	50	65.2 (± 5.0)	66.2 (± 3.4)	UE and LE	FMA, TIS, BBS, TUG, and FRT	Received Physical therapy, occupational therapy, and functional electrical stimulation (FES).	To investigate the preliminary therapeutic efficacy and usefulness of canoe game-based virtual reality training for stroke patients.	Canoe game-based virtual reality training is an acceptable and effective intervention for improving trunk postural stability, balance, and UE motor function in stroke patients
Bergmann, 2018 (46)	Germany	RCT	20	70	62 (± 11)	65 (± 8)	UE	Functional Ambulation Classification, the 10m walk test, a 10-m dual task, the 6min walk test, and muscle strength of the lower extremity using the Medical Research Council Scale	Received 12 sessions (4 weeks, 3 sessions per week) of standard robot-assisted gait training	To evaluate the acceptability of robot-assisted gait training (RAGT) with and without VR and the feasibility of potential outcome measures to guide the planning of a larger randomized controlled trial (RCT).	VR-augmented RAGT resulted in high acceptability and motivation, and in a reduced drop-out rate and an extended training time compared to standard RAGT. This pilot trial provides guidance for a prospective RCT on the effectiveness of VR-augmented RAGT.
Lee, 2018 (23)	Korea	RCT	30	60	61.8 (± 6.8)	61.33 (± 8.44)	UE and LE	The modified FRT, postural sway test, WBB, and MFT	Received a conventional rehabilitation program consisting of physical therapy and occupational therapy	To investigate the effects of game-based VR canoe paddling training, when combined with conventional physical rehabilitation programs, on postural balance and upper extremity function in 30 patients with subacute stroke	Game-based VR canoe paddling training is an effective rehabilitation therapy that enhances postural balance and upper extremity function in patients with subacute stroke when combined with conventional physical rehabilitation programs
Choi, 2014 (47)	Korea	RCT	20	50	64.30 (± 10.3)	64.7 (± 11.3)	UE	FMA for UE, MFT, BBT, and grip strength	Received conventional occupational therapy for 30 minutes a day, five times a week for 4 weeks	To investigate the effectiveness of commercial gaming-based virtual reality (VR) therapy on the recovery of paretic upper extremity in subacute stroke patients	The commercial gaming-based VR therapy was as effective as conventional OT on the recovery of upper extremity motor and daily living function in subacute stroke patients
Brunner, 2017 (48)	Norway	RCT	120	64.16	62	62	UE	ARAT, Abilhand Scale, BBT and FIM	Received exercises for different gross movements and dexterity using a variety of grips and selective finger movements.	To compare the effectiveness of upper extremity virtual reality rehabilitation training (VR) to time-matched conventional training (CT) in the subacute phase after stroke.	Additional upper extremity VR training was not superior but equally as effective as additional CT in the subacute phase after stroke. VR may constitute a motivating training alternative as a supplement to standard rehabilitation
Cannell, 2018 (49)	Australia	RCT	79	51.9	72.8 (± 10.4)	74.8 (± 11.9)	UE and LE	Standing balance (functional reach), lateral reach, timed BBT, step test, sitting balance, arm function, and walking	Participants were scheduled to receive two sessions of therapy per day. Both groups received individually prescribed physical therapy targeting functional outcomes on a daily basis. the second session, participants received individualized prescription of repetitive exercises (functional retraining, strength, balance, and endurance).	To compare the efficacy of novel interactive, motion capture-rehabilitation software to usual care stroke rehabilitation on physical function	No differences between the rehabilitation units were seen except in lateral reach (less affected side) (P = 0.04). No adverse events were recorded during therapy.
Yavuzer, 2008 (50)	Turkey	RCT	20	45	58.1 (± 10.2)	64.1 (± 5.8)	UE	B-stages and the self-care sub-items of FIM	A conventional stroke rehabilitation program, 5 days a week, 2-5 hours/day for 4 weeks. The conventional program is patient-specific and consists of neurodevelopmental facilitation techniques, physiotherapy, occupational therapy, and speech therapy (if needed). The duration of the treatment for UE was approximately 1 hour.	To evaluate the effects of “PlayStation EyeToy Games” on upper extremity motor recovery and upper extremity-related motor functioning of patients with subacute stroke.	“PlayStation EyeToy Games” combined with a conventional stroke rehabilitation program have a potential to enhance upper extremity-related motor functioning in subacute stroke patients.
Wang, 2017 (51)	China	RCT	26	84.61	55.33 (± 8.40)	53.38 (± 7.65)	UE	WMFT and fMRI	Received conventional occupational therapy twice a day, each 45 minutes, 5 days per week for 4 weeks	To evaluate the brain function reorganization by fMRI, as well as the motor function recovery of the affected UE in patients with subacute stroke using Leap Motion-based virtual reality training.	The Leap Motion-based virtual reality training was a promising and feasible supplementary rehabilitation intervention, could facilitate the recovery of motor functions in subacute stroke patients
Turkbey, 2017 (52)	Turkey	RCT	19	73.68	61.7	62.44	UE	Total training time, training time per session, number of sessions, BBT, WMFT, self-care subscale of FIM, and upper extremity BMRS	Received conventional rehabilitation programme consisted of passive and active range of motion exercises, therapeutic stretching, muscle strengthening, neurophysiological exercises, sitting, standing, balance and gait exercises, occupational therapy and activities of daily living training, such as eating, grooming, dressing, toileting and transfer for 4 weeks (60 min/day, 5 days/week)	To evaluate the feasibility and safety of Xbox KinectTM training of the upper extremity in subacute stroke rehabilitation.	Xbox KinectTM training appears feasible and safe in upper extremity rehabilitation after stroke. It could enhance motor and functional recovery of the affected upper extremity as an adjunctive method.
Sheehy, 2020 (61)	Canada	RCT	69	60.87	64.9 (± 15.8)	64.7 (± 16.2)	UE	Function in Sitting Test, Ottawa Sitting Scale, Reaching Performance Scale, and WMFT.	played five games that required limited arm movement and minimal trunk movement, for example reaching within arms' length to virtually pick up cutlery from a table and put it in a drawer, using small arm movements to move a virtual fish along a vertical track.	To determine if supplemental sitting balance exercises, administered via VRT, improve control of sitting balance and upper extremity function in stroke rehabilitation inpatients	Siting balance outcomes were similar for both groups; therefore, this study does not support the use of sitting balance exercises provided via VRT for the rehabilitation of sitting balance after stroke
Simşek, 2016 (53)	Turkey	RCT	42	NA	58.04 (± 16.56)	61.5 (± 0.99)	UE and LE	Turkish translation of the FIM, Visual Analog Scale, and Nottingham Health Profile-NHP	Received conventional treatment (obath neurodevelopmental treatment) for 10 weeks (45–60 hours/day, 3 days/week).	To investigate the effects of Nintendo WiiTM-based balance and upper extremity training on activities of daily living and quality of life in patients with subacute stroke	The Nintendo Wii training was as effective as Bobath NDT on daily living functions and quality of life in subacute stroke patients
Rogers, 2019 (54)	Australia	RCT	21	42.85	64.3 (± 17.4)	64.6 (± 12.0)	UE	MoCA, GMLT and SST from the CogState computerized assessment battery, and NFI	Received 3 h of daily conventional occupational and physiotherapy, provided by the treating allied health rehabilitation service at the hospital	To evaluate the efficacy of Elements as a virtual rehabilitation approach for stroke survivors	A course of Elements virtual rehabilitation using goal-directed and exploratory upper-limb movement tasks facilitates both motor and cognitive recovery after stroke. The magnitude of training effects, maintenance of gains at follow-up, and generalization to daily activities provide compelling preliminary evidence of the power of virtual rehabilitation when applied in a targeted and principled manner
Morone, 2014 (55)	Italy	RCT	50	NA	58.36 (± 9.62)	61.96 (± 10.31)	LE	10 m walk test at a self-selected speed, Functional Ambulatory Category, and BI	Received standard physiotherapy 20 minutes of balance therapy 3 times/week for 4 weeks. In light of the patient's ability, the balance exercises were focused on trunk stabilization, weight transfer to the paretic leg, and exercise with Freeman board for balance and proprioception.	To investigate the efficacy of balance training using video game-based intervention on functional balance and disability in individuals with hemiparesis due to stroke in subacute phase.	Balance training performed with a Wii Fit as an add on to the conventional therapy was found to be more effective than conventional therapy alone in improving balance and reducing disability in patients with subacute stroke.
Brunner, 2016 (56)	Norway	RCT	50	56	59.6 (± 15.6)	61.6 (± 12.6)	UE	ARAT, BBT, and FIM	Received conventional arm training comprised task-related practice for gross movements and dexterity including different grips and selective finger movements, strength training, stretching, and training in daily life activities. Patients in both groups were encouraged to active training.	To compare intensity and content of a VR training intervention to a conventional task-oriented intervention (CT).	Patients with severely impaired UL motor function spent more time actively in VR training, which may influence recovery. The upcoming results of the VIRTUES trial will show whether this is correlated with an increased effect of VR compared to CT.
Iosa, 2015 (57)	Italy	cross-over pilot trial	4	50	NA	NA	UE	The Pittsburgh Rehabilitation Participation Scale, hand ability and grasp force evaluated, respectively, by means of the Abilhand Scale and by means of the dynamometer	Received conventional therapy program, formed by two daily sessions of physiotherapy, each one lasting 40 minutes, 5 days per week	To explore the feasibility of adapting the leap motion controller, developed for videogames, to neurorehabilitation of elderly with subacute stroke.	The leap motion controller can be a suitable tool even for elderly patients with subacute stroke. LMC training was in fact performed with a high level of active participation, without adverse effects, and contributed to increase the recovery of hand abilities
Saposnik, 2010 (12)	Canada	RCT	22	63.63	55.3	67.3	UE	Modified Rankin scale, BI, Canadian Neurological Scale, the Stroke Impact Scale, and The Borg perceived exertion scale	Received recreational therapy sessions included leisure activities such as playing cards, stamping a seal while playing bingo, or playing Jenga.	To examine the feasibility and safety of the VR Nintendo Wii gaming system (VRWii) compared with recreational therapy (RT) in facilitating motor function of the upper extremity required for activities of daily living among patients with subacute stroke receiving standard rehabilitation	Virtual reality using the Nintendo Wii gaming system gaming technology represents a safe, feasible, and potentially effective alternative to facilitate rehabilitation therapy and promote motor recovery after stroke

*RCT, randomized controlled trial; VR, virtual reality; fMRI, functional magnetic resonance; FMA, fugl-meyer assessment; BBT, box and block test, B-Stage, brunnstrom stage; BI, barthel index; TAC, total activity count; UE, upper extremity; LE, lower extremity; FMI, functional independence measure; ARAT, action research arm test; MFT, manual function test; TIS, trunk impairment scale; BBS, berg balance scale; TUG, timed up-and-go tests; FRT, functional reach test; WBB, wii balance board; MoCA, montreal cognitive assessment; GMLT, groton maze learning task; SST, set shift task; NFI, neurobehavioural functioning inventory; and WMFT, the wolf motor function test*.

### Risk of Bias

**Figure 2:**
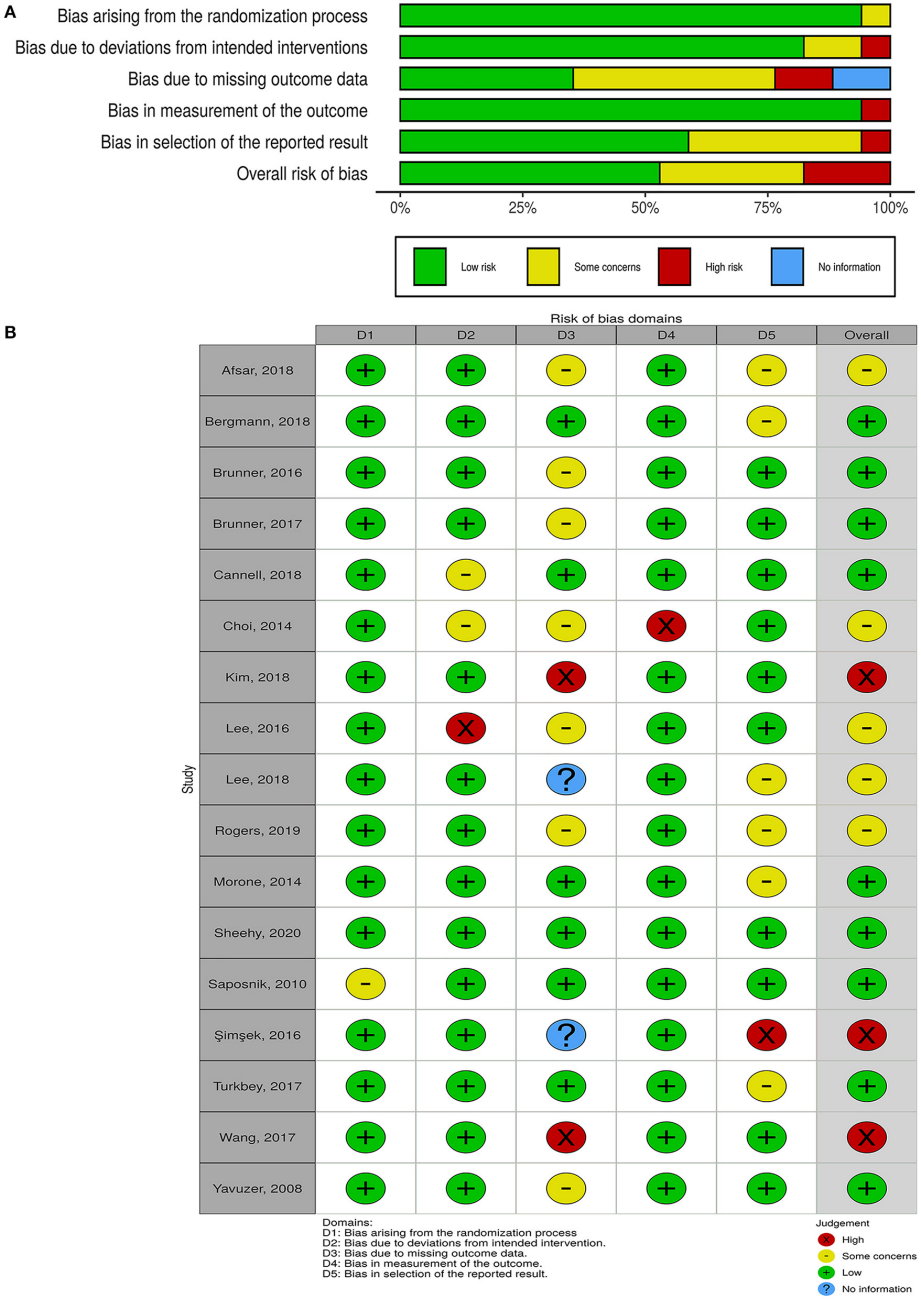
Quality of the included randomized controlled trials. **(A)** Risk-of-bias graph: review authors' judgments about each risk-of-bias item presented as percentages across all included studies. **(B)** Risk-of-bias summary: review authors' judgments about each risk-of-bias item for each included study.

**Figure 3:**
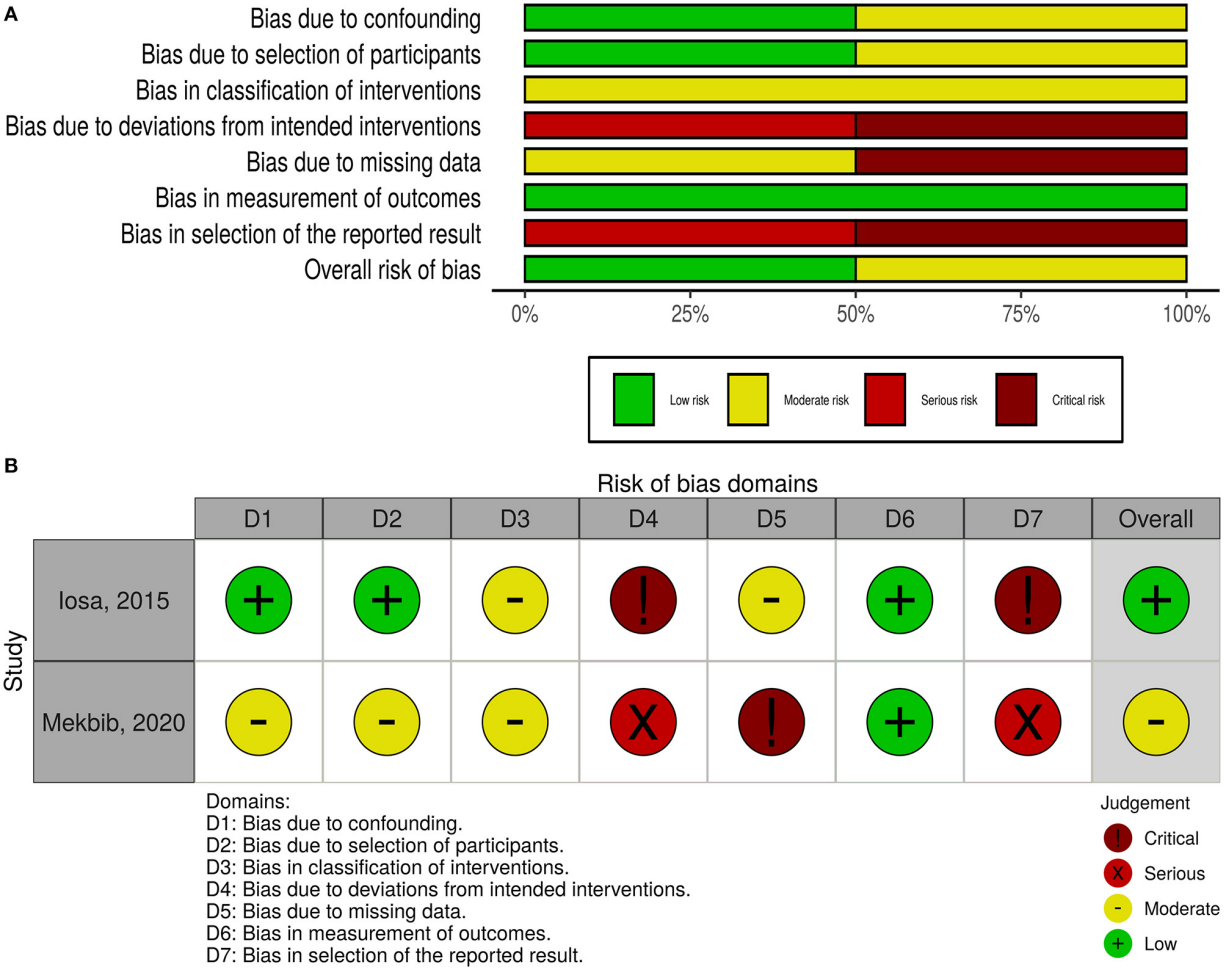
Quality of the included non-randomized controlled trials. **(A)** Risk-of-bias graph: review authors' judgments about each risk-of-bias item presented as percentages across all included studies. **(B)** Risk-of-bias summary: review authors' judgments about each risk-of-bias item for each included study.

The overall risk of bias was low in nine studies, whereas five studies had some concerns, and three had a high risk of bias (Figure 2). For non-randomized trials, the risk of bias was moderate in one of them and low in the other (Figure 3).

### Effectiveness of VR Therapy

**Figure 4:**
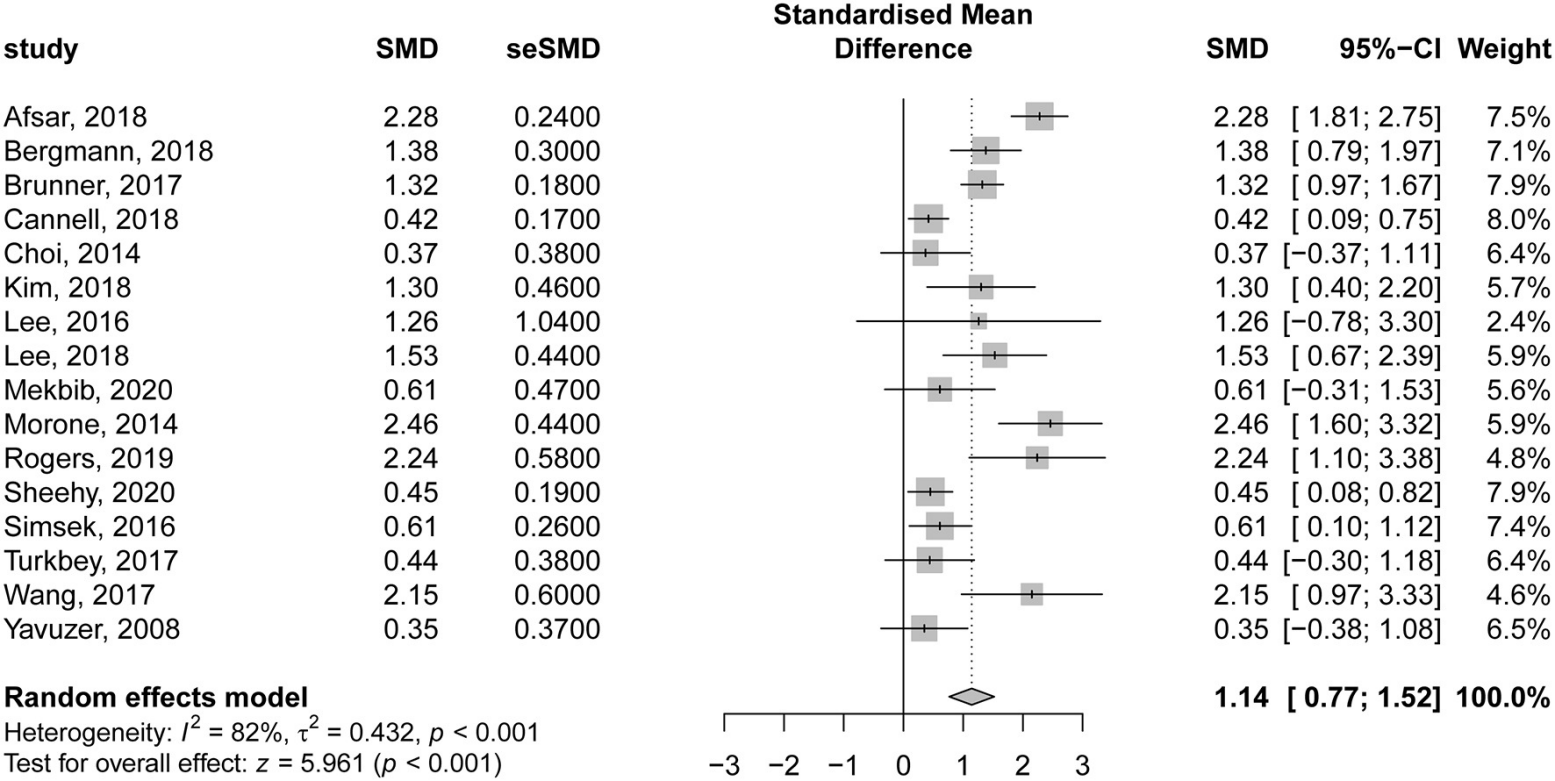
Forest plot for the effectiveness of virtual reality–based rehabilitation of motor function in subacute stroke patients (preintervention vs. postintervention).

The details of VR-based interventions and the control groups are provided in Table 2. Sixteen studies evaluating the effectiveness of VR therapy (preintervention vs. post intervention) were included in the analysis. There was a significant improvement in motor function score following VR therapy in patients with subacute stroke, compared to their preintervention score (SMD = 1.14; 95% CI = 0.77–1.52; *P* < 0.001). According to Egger regression test, there was no significant risk of bias among included studies (*P* = 0.275); however, significant heterogeneity was present (*I*^2^ = 82% and *P* < 0.001) (Figure 4). The contribution of each study to the overall heterogeneity is presented in Supplementary Figure 1. Moreover, the leave-one-out sensitivity analysis did not affect the significance of the overall effect size, indicating that the findings were not driven by any single study Supplementary Figure 2. The meta regression analysis did not show any significant effect of the post stroke duration (days) on the treatment effects (*P* = 0.230) (Supplementary Figure 3).

**Table 2:** Description of the experimental and control groups of included studies.

**Author, year**	**Intervention Group**	**Control Group**		
	**Description**	Duration^*^	**Description**	Duration^*^
Mekbib, 2020 (44)	This group received: (1) conventional training (same as control) and (2) MNVR therapy. The MNVR-Rehab system comprises of the following elements: (1) A HTC Vive head-mounted display (HMD) to fully immerse the patient in the virtual environment; (2) two base stations (lighthouses) to track the patient's exact location in 3D; (3) a piece of Leap Motion to track the patient's UE movements and transfer the movements onto a virtual limb in the virtual environment; and (4) a high-performance PC with powerful graphics as the central controller, running the software system to generate the virtual environment, supervise the participant's performance, record the patient's actions, and choose various training options. This system provides game-based exercises of unilateral and bilateral reach-to-grasp tasks.	1 h x 4 days x 2 weeks	This group received conventional therapies (not clearly described).	1 h x 4 days x 2 weeks
Afsar, 2018 (45)	This group received conventional therapy (1 hour per session) in addition to VR training using Xbox Kinect (1/2 hour per session) using the following games: Mouse Mayhem, Traffic Control, Balloon Buster, and Mathercising from Dr. Kawashima's Body and Brain Exercises package. Patients actively performed bilateral shoulder abduction and adduction, and active elbow flexion and extension movements in the “Mouse Mayhem” and “Traffic Control” games. They also performed flexion and extension movements in both the shoulder and elbow joints in the “Balloon Buster” and “Mathercising” games	0.5 h x 5 days x 4 weeks	This group received conventional therapy (1 hour per session). Physical therapy included static and dynamic control of position, balance skills, weight shift, and activities of daily living	1 h x 5 days x 4 weeks
Kim, 2018 (22)	This group received: (1) occupational therapy (0.5 hours /5 days/2 weeks) and (2) kinetic-based VR rehabilitation system. This system three types of programs: “Push Museum,” “Apple Run,” and “Fruit Market.” These programs were made using the Unity three-dimensional (3D) game engine (Unity Technology Inc., San Francisco, CA). This system induces arm motions important during rehabilitation (reaching, wrist extension, hand grasping, and releasing).	0.5 h x 5 days x 2 weeks	This group received: (1) occupational therapy (0.5 hour/5 days/2 weeks) and (2) Sham VR rehabilitation. A similar strategy as for real VR was applied and subjects were instructed to use the hemiparetic upper limb. In this group patients reach to the button and pushing the selected button during the cognitive task.	0.5 h x 5 days x 2 weeks
Lee, 2016 (21)	This group received: (1) conventional rehabilitation program (same as control) and (2) canoe game-based VR rehabilitation therapy. The canoe game-based VR training program was conducted using the Nintendo Wii Sports Resort package. Participants paddled by grasping the motion controller, alternating between hands while sitting on the springboard. They also adjusted their trunk to maintain balance on the springboard during paddling.	0.5 h x 3 days x 4 weeks	This group received: conventional rehab program alone. The conventional program consists of: physical therapy for gait training and lower limb strengthening (0.5 h twice daily/5 days/4 weeks), occupational therapy to improve performance in activities of daily living (0.5 hour twice daily/5 days/4 weeks), and FES applied to both UL and LL (15 min/5 days/4 weeks).	Variable
Bergmann, 2018 (46)	This group received: (1) RAGT (standard protocol) and (2) VR training: using two VR scenarios (the coin scenario and the dog scenario)- both scenarios took place in a forest, where subjects had to walk along a straight alley in the middle of the screen, and solve different tasks by controlling the avatar's speed by adapting their motor activity. In the dog scenario, patients' activity was displayed as a red dot on the path, where the patient is instructed to place the red dot underneath the dog. In the coin scenario, coin scenario, patients had to collect coins, and avoid rocks that were placed on the path. The dog scenario was applied in sessions 1&2, while the coin scenario was applied in sessions 3&4. Furthermore, this group received physiotherapy sessions (1 hour x 2 days x 4 weeks).	1 h x 3 days x 4 weeks	Standard RAGT using the robotic-driven gait orthosis lokomat. Patients were fixed into the gait orthosis with a harness, which was attached to a body-weight support system, and had cuffs placed around the legs. Furthermore, this group received physiotherapy sessions (1 h x 2 days x 4 weeks).	1 h x 3 days x 4 weeks
Lee, 2018 (23)	This group received: (1) conventional physical therapy (same as control) + (2) game-based VR canoe paddling training using the Nintendo Wii Sports Resort game. Patients performed a paddling movement with both hands grasping the motion controller that was inserted in a separate canoe paddle accessory. Participants operated the paddle in the direction of the virtual character displayed on an LED TV 42LN549C screen. They were also instructed to focus on trunk control to maintain their balance on top of the springboard, while canoe paddling.	0.5 h x 3 days x 5 weeks	This group received conventional physical therapy in the form of: (1) physical therapy: to improve balance and lower limb strength to facilitate walking (0.5 h/session/twice a day) and (2) occupational therapy: to improve the performance of activities of daily living (0.5 h/session/twice a day)	2 h x 5 days x 5 weeks
Choi, 2014 (47)	This group received: (1) conventional rehabilitation therapy and (2) gaming-based VR movement therapy: using the Wii (Nintendo) which consists of 12 games; however, only 3 games were chosen (the swordplay, table tennis, and canoe games). The swordplay game involved performing flexion, extension, internal and external rotation of the shoulder, and flexion and extension of the elbow. The table tennis and canoe games also required upper extremity motions including internal and external rotation of the shoulder, flexion and extension of the elbow, and pronation and supination of the forearm.	0.5 h x 5 days x 4 weeks	This group received only conventional occupational therapy in the form of highly repetitive trainings. It composed of stretching and strengthening exercises using full range of motion of the upper extremity, which was a task-oriented therapy for the ADL, fine motor training, and sensory motor recovery.	0.5 h x 5 days x 4 weeks
Brunner, 2017 (48)	This group received: (1) VR training using the You Grabber system which contains several games; The different therapy modes include reaching and grasping exercises, selective finger movements, supination/pronation, whole-arm movements, unimanual or bimanual training, and virtually enhanced movements, i.e., movements that can be visually increased on the screen + (2) standard rehabilitation (individually-based).	1 h x (4–5) days x 12 weeks	This group received: (1) conventional training which included exercises for different gross movements and dexterity using a variety of grips and selective finger movements + (2) standard rehabilitation (individually-based)	1 h x (4–5) days x 12 weeks
Cannell, 2018 (49)	This group received two therapy sessions per day for five days/week for 8 weeks. Each of these sessions were 1 hour long. The first session involved individually-prescribed physical therapy (1 hour/5 days/ 8 weeks) and the second session involved individualized prescription of repetitive exercises using the Jintronix Rehabilitation System™ (JRS WAVE). These game-based exercises included: arm activities, sitting and standing tasks, seated and standing leg activities)	1 h x 5 days x 8 weeks	This group received two therapy sessions per day for five days/week for 8 weeks. Each of these sessions were 1 hour long. The first session involved individually-prescribed physical therapy and the second session involved individualized prescription of repetitive exercises (in seated and standing positions)	2 therapy sessions (each is 1 h) x 5 days x 8 weeks
Yavuzer, 2008 (50)	This group received: (1) conventional therapy (2–5 h x 5 days x 4 weeks) and (2) VR rehabilitation using the PlayStation Eye Toy Games. This therapeutic program included flexion and extension of the paretic shoulder, elbow and wrist in addition to abduction of the shoulder.	0.5 h x 5 days x 4 weeks	This group received: (1) conventional therapy (2–5 hours x 5 days x 4 weeks) and (2) placebo in the form of watching games for the same duration without being involved in any kind of physical activity.	0.5 h x 5 days x 4 weeks
Wang, 2017 (51)	This group received: (1) conventional rehab program (same as control) and (2) Leap Motion-based VR training, which consisted of a computer and a leap motion controller. The controller can track, with sub-millimeter accuracy, the movement of multiple hands and fingers. These games were designed to focus on the development of the pinching, grasping, and individuating motor skills of fingers; the improvement of the dexterity and coordination of the digits; the improvement of the ability to flex and extend the hand, the pronation and supination of the forearm; the increase in the joint range of motion of the hand, elbow, shoulder and wrist; the improvement of the movement speed, muscle strength, and motor control.	45 min x 5 days x 4 weeks	This group received conventional rehab program only, which included stretches, strength, balance, gait, and functional training.	1.5 h x 5 days x 4 weeks
Turkbey, 2017 (52)	This group received: (1) conventional rehabilitation program (same as control) and (2) VR rehab program by using the Xbox Kinect TM games console. Activities were performed in a sitting position 2.25–2.75 m from the television screen. Activities involved active flexion, extension, internal and external rotation of shoulder, as well as active elbow flexion and extension of the affected limb.	1 h x 5 days x 4 weeks	This group received conventional rehabilitation program alone. It consists of passive and active range of motion exercises, therapeutic stretching, muscle strengthening, neurophysiological exercises, sitting, standing, balance and gait exercises, occupational therapy and activities of daily living training.	1 h x 5 days x 4 weeks
Sheehy, 2020 (61)	This group received: (1) conventional rehab program (same as control) and (2) VR training using Jintronix software and a Kinect 2 three-dimensional motion-tracking camera. Participants in this training played six Jintronix games that required trunk lean and reaching beyond arms' length.	30–45 min x 5 days x 2 weeks	This group received: (1) conventional rehab program (2–3 sessions a day of physiotherapy, occupational therapy, rehabilitative exercise, and speech-language pathology and (2) placebo. Patients in the placebo arm played five games that required limited arm movement and minimal trunk movement. To minimize trunk movement, participants in the control group sat in a wheelchair with a softer, contoured cushion, with armrests and seatbelt in place and diagonal straps positioned snuggly across the chest.	30–45 min x 5 days x 2 weeks
Simşek, 2016 (53)	This group received Nintendo Wii-based VR training using multiple video games. Patients in this group used five games selected from the Wii sports and Wii Fit packages for upper limbs (tennis and punch out) and balance training (tightrope tension, tilt table and heading), respectively.	45–60 min x 3 days x 10 weeks	This group received conventional therapy in the form of NDT. NDT exercises were done in the bed, in sitting and standing positions. Scapular mobilization, exercises, M. latissumus dorsi stretching, weight shifting to the affected upper extremity, selective strengthening of shoulder stabilizators were done for upper extremity.	45–60 min x 3 days x 10 week
Rogers, 2019 (54)	This group received: (1) conventional occupational and physical therapy and (2) Element VR training using four hand-held objects (i.e., the four “elements” in the shape of a circle, pentagon, triangle, and rectangle), the participant engaged with a virtual environment presented on a 42 in. touchscreen LCD panel (Multitaction™) with inbuilt CPU. Elements tasks included: Task 1 (Bases) consists of the home base and four potential movement targets, all 78 mm in diameter; Task 2 (Random Bases) has the same configuration of targets; Task 3 (Chase Task) begins with a blank screen; Task 4 (Go/No-Go) uses the same target positions as Task 3, however, additional distractor targets (a pentagon, triangle and rectangle) appear; Tasks 5, 6 and 7 require participants to explore the virtual environment, by creating various shapes and sounds through movement.	30–40 min/3 days/4 weeks	This group received: conventional occupational and physical therapy (3 h daily), which was individually-based. This training was focused on range of motion exercises, muscle strengthening and coordination, and re-training of daily living skills.	3 h/day
Morone, 2014 (55)	This group received: (1) conventional physical therapy (same as control and (2) video game-based VR therapy using the Wii Fit. Three games were carried out in order to train balance, coordination, and endurance.	20 min/3 days/4 weeks	This group received: (1) conventional physical therapy (40 min twice a day) and (2) standard balance therapy which focused on trunk stabilization, weight transfer to the paretic leg, and exercise with Freeman board for balance and proprioception	20 min/3 days/4 weeks
Brunner, 2016 (56)	This group received VR training. Patients were seated at a table during the training session and received individually tailored exercises for arm and hand movements according to their needs and abilities. VR training was conducted using the YouGrabber system.	45–60 min x 4–5 days x 4 weeks	This group received conventional training which consisted of task-related practice for gross movements and dexterity including different grips and selective finger movements, strength training, stretching, and training in daily life activities.	45–60 min x 4–5 days x 4 weeks
Iosa, 2015 (57)	This group received: (1) conventional therapy (six sessions, same as control) and (2) leap motion-controlled video game-based VR training. During each session, patient sat in a front of a table on which there was a 27-inch monitor at a distance of about 0.80 m.	0.5 h x 3 days x 2 weeks	This group received conventional therapy in the form of two daily sessions of physiotherapy, each one lasting 40 min, 5 days per week. One daily session was dedicated to arm and hand training, focused on the facilitation of movements on the paretic side, upper-limb exercises for reaching and grasping and for improving proprioception. The second daily physiotherapy aimed to improve balance, trunk stabilization, standing, weight transfer, sitting, transferring, and when possible walking.	2 sessions (40 min each) x 5 days x 2 weeks
Saposnik, 2010 (12)	This group received: (1) standard therapy (not described) and (2) Nintendo Wii game-based VR training. The arm movements involved in the use of the Wii included shoulder flexion and extension (bowling and tennis), shoulder rotation (tennis), elbow extension and flexion (Cooking Mama), wrist supination and pronation (tennis and Cooking Mama), and different degrees of wrist flexion and extension as well as thumb flexion involved in all activities.	1 h x 4 days x 2 weeks	This group received: (1) standard therapy (not described) and (2) recreational therapy in the form of leisure activities such as playing cards, stamping a seal while playing bingo, or playing Jenga.	1 h x 4 days x 2 weeks

* *The duration presented in this table refers to the duration of the VR rehabilitation therapy sessions alone; other conjunctive therapies are described as well. RAGT, robot-assisted gait training; VR, virtual reality; ND, not described; FES, functional electrical stimulation; UL, upper limb; LL, lower limb; MNVR, mirroring neuron virtual reality; NDT, neurodevelopmental treatment*.

**Figure 5:**
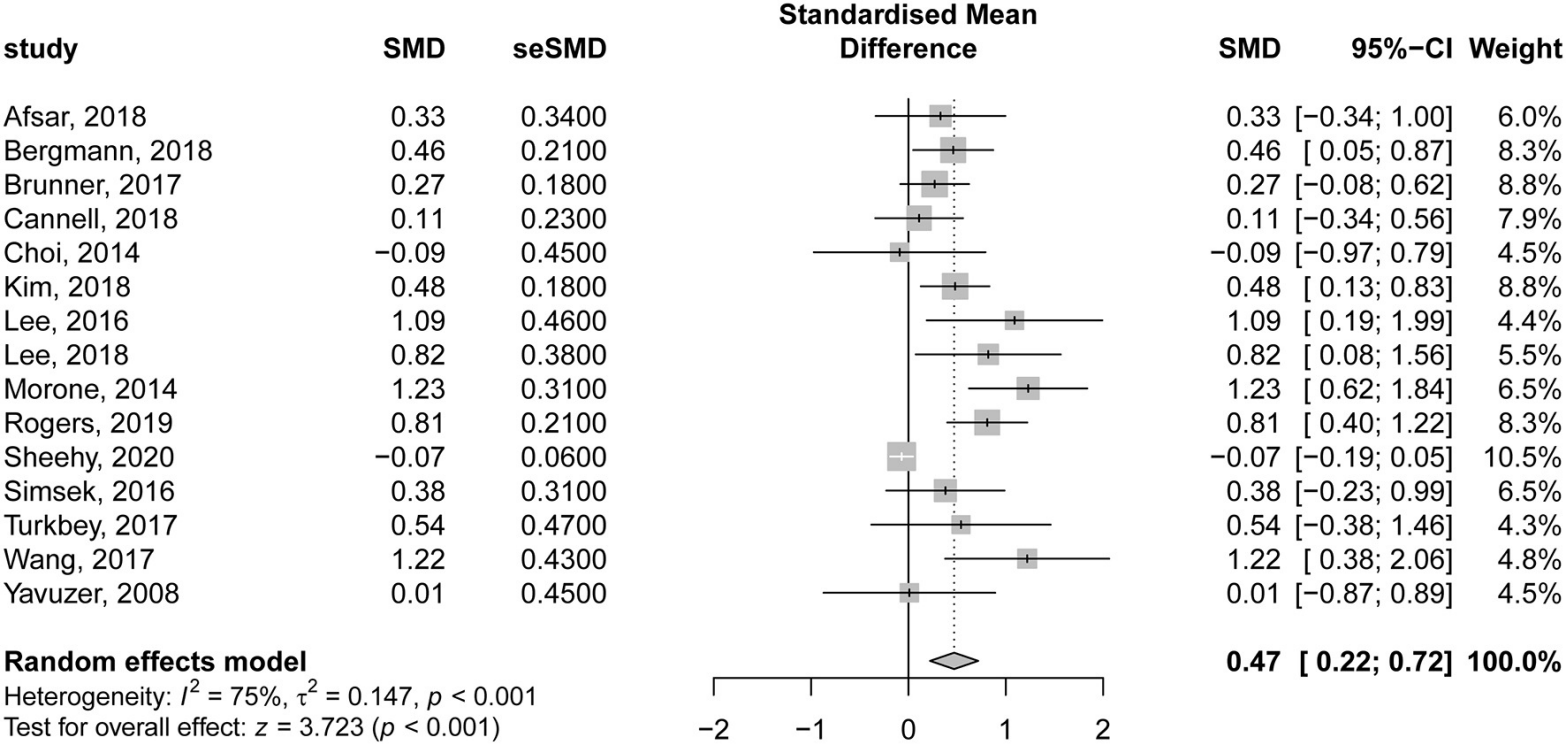
Forest plot for the comparative effectiveness of virtual reality–based rehabilitation of motor function vs. conventional therapy in subacute stroke patients.

In the same context, 15 studies evaluating the comparative effectiveness of VR therapy and CTs were included in the analysis. There was a significant improvement in motor function following VR therapy in patients with subacute stroke, compared to those undergoing CT (SMD = 0.47; 95% CI = 0.22–0.72; *P* < 0.001). Nevertheless, there was significant heterogeneity among the included studies (*I*^2^ = 75% and *P* < 0.001) (Figure 5). The contribution of each study to the overall heterogeneity is presented in Supplementary Figure 4. The leave-one-out sensitivity analysis did not affect the significance of the overall effects indicating that the findings were not driven by a single study (Supplementary Figure 5).

**Figure 6:**
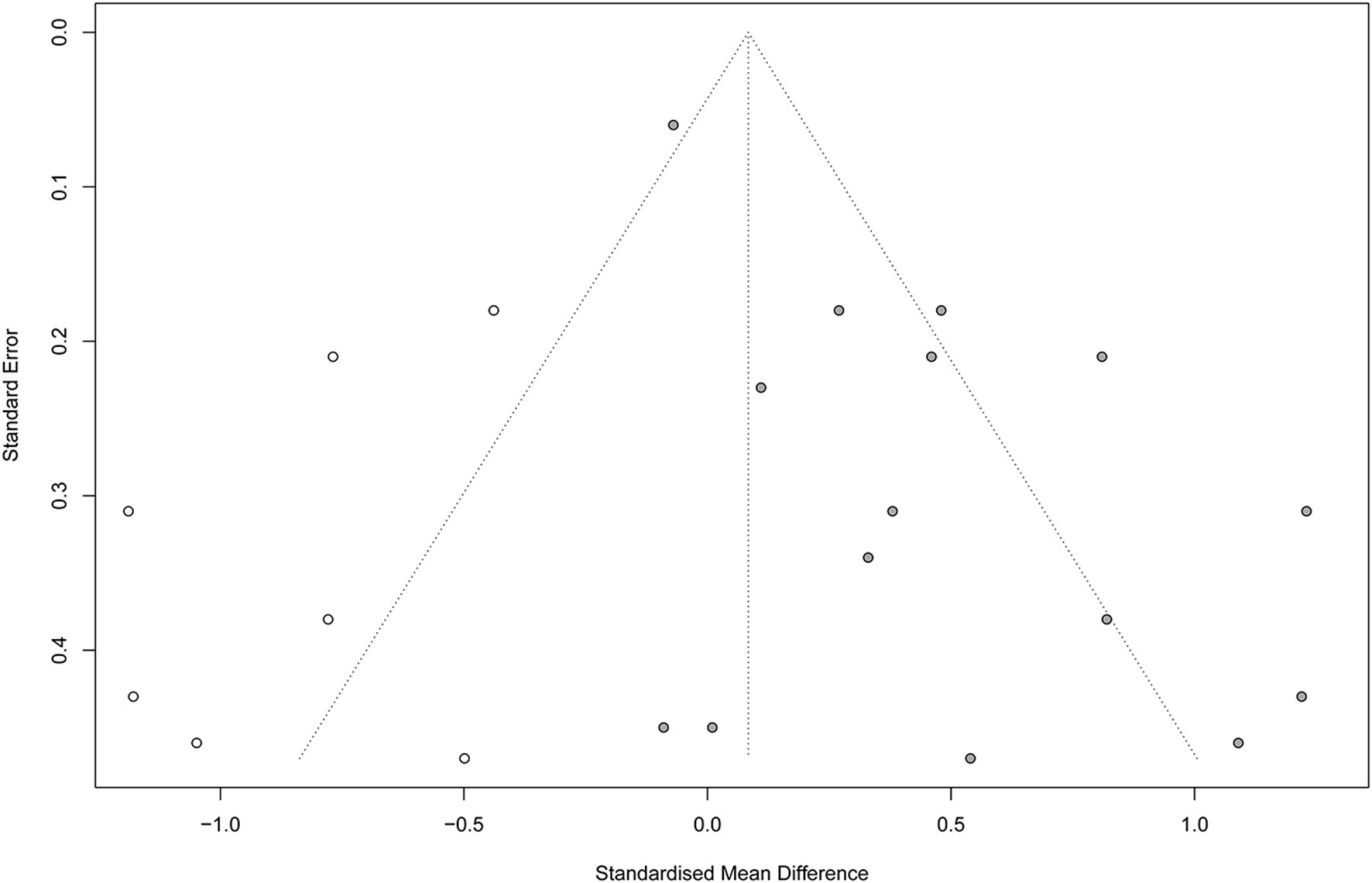
Funnel plot with trim-and-fill method. Number of studies combined: *k* = 22 (with seven added studies).

Noteworthy, according to Egger regression test, there was a significant risk of bias among the included studies (*P* = 0.001). Therefore, we used the trim-and-fill method to “trim” the studies that caused the asymmetry of the funnel plot so that the overall effect estimate would be minimally affected by publication bias and then to “fill” imputed missing studies in the plot according to the bias-corrected overall effect estimate. Unfortunately, upon using this method, the overall effect estimate was deemed insignificant (SMD = 0.08; 95% CI = −0.16–0.33; *P* = 0.507), with a higher heterogeneity (*I*^2^ = 82.6% and *P* < 0.001) (Figure 6). This indicates that VR therapy is not associated with significant improvement in motor function as compared with CT. The meta regression analysis did not show any significant effect of the post stroke duration (days) on the treatment effects (*P* = 0.413) (Supplementary Figure 6).

## Discussion

The field of neurorehabilitation after stroke, especially during the subacute phase, is still evolving. The current paradigms of neurorehabilitation strategies in order to enhance the motor function of affected extremities are focused on high-intensity, task-specific interventions with the main character of repetition (58, 59). The repetition of such programs or tasks potentiates the acquisition of new along with the retrieval of previous motor functions of affected limbs, leading to long-term potentiation. VR game–based programs are a group of interventions that are directed to incorporate affected populations in a computer-simulated environment while giving them almost real-time feedback on their performance. According to previous evidence (59, 60), the degree to which VR programs can aid or facilitate current CT programs is still to be determined.

We conducted this meta-analysis to determine the comparative effectiveness of VR-based systems in the rehabilitation of the motor function of affected limbs in patients with subacute stroke. We aimed to investigate the effect of VR on restoring the motor function of both UL and LL; however, this was not possible because the majority of included studies investigated the effect of VR on the motor function of the upper limbs, whereas only two studies recruited patients with hemiparesis with no description of which limb was investigated. Therefore, we reported the change in the motor function of affected limbs, with no discrimination, following VR-based therapy as compared to CT. The CT group included various programs: physical therapy, occupational therapy, functional electrical stimulation, or a combination of them.

In our study, we noted that VR interventions resulted in a great improvement in motor function of affected limbs in patients with subacute stroke compared to preintervention values (SMD = 1.14; 95% CI = 0.77–1.52). However, we detected significant considerable heterogeneity, which on further analysis revealed that the study of Afsar et al. (45) was the major contributor to the resultant heterogeneity. This could be related to their heterogeneous population compared to other studies. They included patients with mild to moderate upper limb motor deficits, and they were further subdivided into two groups (VR vs. conventional). Furthermore, patients in both groups received CT. Also, patients in the VR group received mild training of 30 min per day 5 days a week for 4 weeks using the Xbox Kinect gaming console in addition to CT of 60 min five times a week for 4 weeks. The aforementioned factors could contribute to the observed heterogeneity. Furthermore, the sensitivity analysis revealed that the effect size “great improvement” was not driven by a single study. The variable “time since last stroke” had no significant effect on the outcomes.

Upon comparing VR programs to CT, our analysis revealed a significant mild improvement (0.5 ≤ SMD ≤ 0.8) in the motor function of the affected limb in favor of VR-based rehabilitation programs. We observed considerable heterogeneity, and the study of (61) was the major contributor. This could be explained by the fact that both groups (VR and control group) in the previous study had VR training of the upper limb in addition to their assigned interventions. Surprisingly, following the trim-and-fill adjustment of risk of bias, the observed results were deemed insignificant (SMD = 0.08; 95% CI = −0.16 to 0.33), indicating no significant difference between both interventions on restoring the motor function of affected limbs. Our findings are comparable to the systematic review and meta-analysis of Laver et al. (62), who assessed the efficacy of VR in improving upper limb motor function in patients with stroke. A significant but mild improvement (SMD = 0.28) in motor function was noted in favor of VR-based interventions. Noteworthy, only 2 of the 12 analyzed studies recruited patients with subacute stroke (9, 12). Therefore, our study provides stronger evidence in regard to patients with subacute stroke, indicating that VR does not result in significant improvement in motor function. In 2019, an umbrella review of meta-analyses was conducted to determine the impact of various neurorehabilitation interventions on changes in ADLs in patients with subacute stroke (63). A total of 55 meta-analyses were investigated, reporting 21 subacute rehabilitation interventions. Of investigated interventions, VR was reported by two meta-analyses (62, 64) and resulted in mild to moderate improvement of ADLs in the subacute phase of stroke rehabilitation. However, the authors highlighted that the lack of high-quality evidence in analyzed meta-analyses highlights the need for more research. In our review, the majority of studied interventions were designed mainly to improve motor function rather than other outcomes, including cognitive function, activity performance, ADLs, or feasibility. As for rehabilitation of motor function, it was reported that constraint-induced motor therapy is considered, by far, the most promising intervention in patients with stroke, in general, based on the findings of a recent systematic review (59).

The feasibility of VR-based rehabilitative interventions was assessed in three trials. In the crossover trial of Iosa et al. (57), four elderly patients with subacute stroke were allocated to receive six sessions of 30 min of leap motion controller–based intervention in addition to CT. Participation in these sessions was excellent in three patients and very good in the remaining patient. This highlights the feasibility of this intervention for neurorehabilitation in this patient group due to the easiness of its use without the need for the subject to stand alone. However, because of the limited number of included participants, these conclusions still need confirmation by larger, well-conducted trials. In the pilot, randomized controlled trial of (12), patients were allocated to receive Nintendo Wii gaming (nine patients) or recreational therapy in the form of playing cards, bingo, or Jenga (eight patients). Feasibility was reflected by the total time of intervention receipt. The mean total time of VR was comparable to that of recreational therapy (388 vs. 364 min; *P* = 0.75). Meanwhile, Brunner et al. (56) recorded 50 videos of patients with subacute stroke who were allocated to receive either VR or CT, and the authors reported higher feasibility in the VR group, with higher mean time of active practice (77.6 min) compared to the CT group (67.3 min). Of note, the fact that patients knew they were being recorded affects the validity and interpretation of this finding. Therefore, despite the promising feasibility of VR-based interventions for the neurorehabilitation in patients with subacute stroke, more robust trials of larger sample sizes are still warranted to reach a more definitive conclusion.

In view of the paucity of well-designed randomized controlled trials, the limited funding for stroke rehabilitation programs and research, and the limitations of CT, VR interventions can be used adjunctly with CT for the following reasons: (1) VR-based interventions are accessible for all patients with subacute stroke, (2) the low cost of VR programs, and (3) it requires no special resources or assistance. Therefore, we hypothesize that VR interventions might potentiate motor rehabilitation in subacute stroke patients when added to CT. However, this finding remains inconclusive for the following reasons.

First, a wide variety of VR programs were reported. Each of these programs included different activities aiming to improve the function of certain parts of the affected limb. For example, the VR program in the study of (45) included the active abduction, adduction, flexion, and extension of the shoulder in addition to flexion and extension of the elbow. Meanwhile, Choi et al. (47) focused on regaining the motor function of affected ULs by promoting certain activities in their VR program, including extension and internal and external rotation of the shoulder; flexion and extension of the elbow; and pronation and supination of the forearm. On the other hand, another trial focused on regaining more delicate motor functions, and therefore the VR programs were more focused on improving the delicate movements of the fingers (promoting the development of pinching and grasping), digits (improving their dexterity and coordination), and hand (enhancing flexion and extension) (51). Other VR programs were primarily used to regain arm motions, such as reaching, wrist extension, and hand grasping and releasing (22).

Another point worth mentioning is the differences in VR programs among included trials. Typically, VR interventions include a head-mounted display, which allows users to experience 3D content (either videos or games) in an immersive virtual environment. In the case of VR interventions in stroke patients with affected ULs, leap motions are usually used in order to track the user's movements. Three of the included trials in our review used this method in their treatment protocol to regain the motor function of affected limbs in patients with subacute stroke (44, 51, 57). However, the remaining studies used the terms “virtual reality intervention” and “game-based intervention” interchangeably. For instance, their treatment protocols incorporated the use of different gaming-based engines or systems, such as the Xbox Kinect system (45, 52), Unity 3D game engine (22), Nintendo Wii Sports Resort package (12, 21, 23, 47, 53), the YouGrabber system (56), Jintronix Rehabilitation system (49), and PlayStation console (50).

Of note, the intensity of the VR intervention was widely variable among included studies. For example, in the study of Brunner et al. (56), a total of 62 patients with subacute stroke underwent VR therapy using the YouGrabber gaming system. The VR therapy lasted for 1 h each and was performed four to five times a week for 12 consecutive weeks. On the other hand, the intensity of the VR intervention was low in the study of (55), where patients with subacute stroke underwent video game–based VR therapy sessions that lasted 20 min each. These sessions were repeated three times per week for a total of 4 consecutive weeks. Therefore, we could not determine the effect of VR intensity on our outcome of interest.

The aforementioned points reflect the heterogeneity of studied populations at baseline, which could also explain the reason for encountering the highly significant considerable heterogeneity in our analyses.

Although our study is the first study to investigate the efficacy of VR interventions in restoring the motor function of patients with subacute stroke, several limitations were encountered. First, most included studies compared VR programs in addition to CT compared to CT alone, which subsequently accounted for more rehabilitation time in the experimental group. This could potentially account for a bias in favor of VR therapy because both the intensity and frequency of rehabilitation, *per se*, are known to be directly correlated with beneficial functional outcomes. Second, the number of participants in included studies was small, ranging from 4 to 120 patients, and thus, the generalizability of our findings could be affected. Third, eight randomized controlled trials had some concerns and a high risk of bias. Based on these limitations, we recommend conducting more robust, well-designed trials of larger sample sizes and longer follow-up periods to reach more definitive conclusions. Fourth, outcomes were assessed directly after the application of the VR interventions with a short follow-up period, ranging from 2 to 12 weeks (44, 56). Therefore, we are not confident that the improvement in motor function following the implementation of VR protocols is long-lasting. Eventually, our systematic review was conducted mainly to determine the effect of VR intervention on the motor function of patients with subacute stroke. Only two articles reported outcomes related to the cognitive function of subacute stroke patients following VR therapy; however, these studies used different measurement tools with different cutoff values for defining cognitive impairment (47, 54). Therefore, it was inapplicable to conduct a meta-analysis on this outcome, and thus, more research is needed to determine the impact of these VR interventions on other outcomes, such as cognitive function, ADLs, and quality of life.

VR provides a great improvement in motor function in patients with subacute stroke, compared to the preintervention state. However, when compared to CT, mild to no significant improvement in motor function was noted. Therefore, VR can be used as an adjuvant to CT in restoring the motor function of affected limbs. That being said, more studies are still warranted to reach a more definitive conclusion and to investigate the effect of VR on the cognitive function and physical performance of affected patients.

## Data Availability Statement

The original contributions presented in the study are included in the article/Supplementary Material, further inquiries can be directed to the corresponding authors.

## Author Contributions

Q-cP and LY wrote the manuscript. Q-cP, LY, and YC collected and analyzed the data. All authors contributed to the article and approved the submitted version.

## Conflict of Interest

The authors declare that the research was conducted in the absence of any commercial or financial relationships that could be construed as a potential conflict of interest.

## Supplementary Material

Supplementary Figure 1 Baujat plot showing the contribution of each study into the overall heterogeneity.

Supplementary Figure 2 Leave-one-out sensitivity analysis for the effectiveness of virtual reality–based rehabilitation (preintervention vs. postintervention).

Supplementary Figure 3 Metaregression of the poststroke duration and virtual reality–based rehabilitation effectiveness (preintervention vs. postintervention).

Supplementary Figure 4 Baujat plot showing the contribution of each study into the overall heterogeneity.

Supplementary Figure 5 Leave-one-out sensitivity analysis for the effectiveness of virtual reality–based rehabilitation (VR vs. conventional therapy).

Supplementary Figure 6 Metaregression of the poststroke duration and virtual reality–based rehabilitation effectiveness (VR vs. conventional therapy).
