# Supplementary figures and images for: Effectiveness of Virtual Reality in the Rehabilitation of Motor Function of Patients With Subacute Stroke: A Meta-Analysis
Source: Front Neurol. 2021 May 5;12:639535. doi: 10.3389/fneur.2021.639535 (PMC8131676; doi:10.3389/fneur.2021.639535)

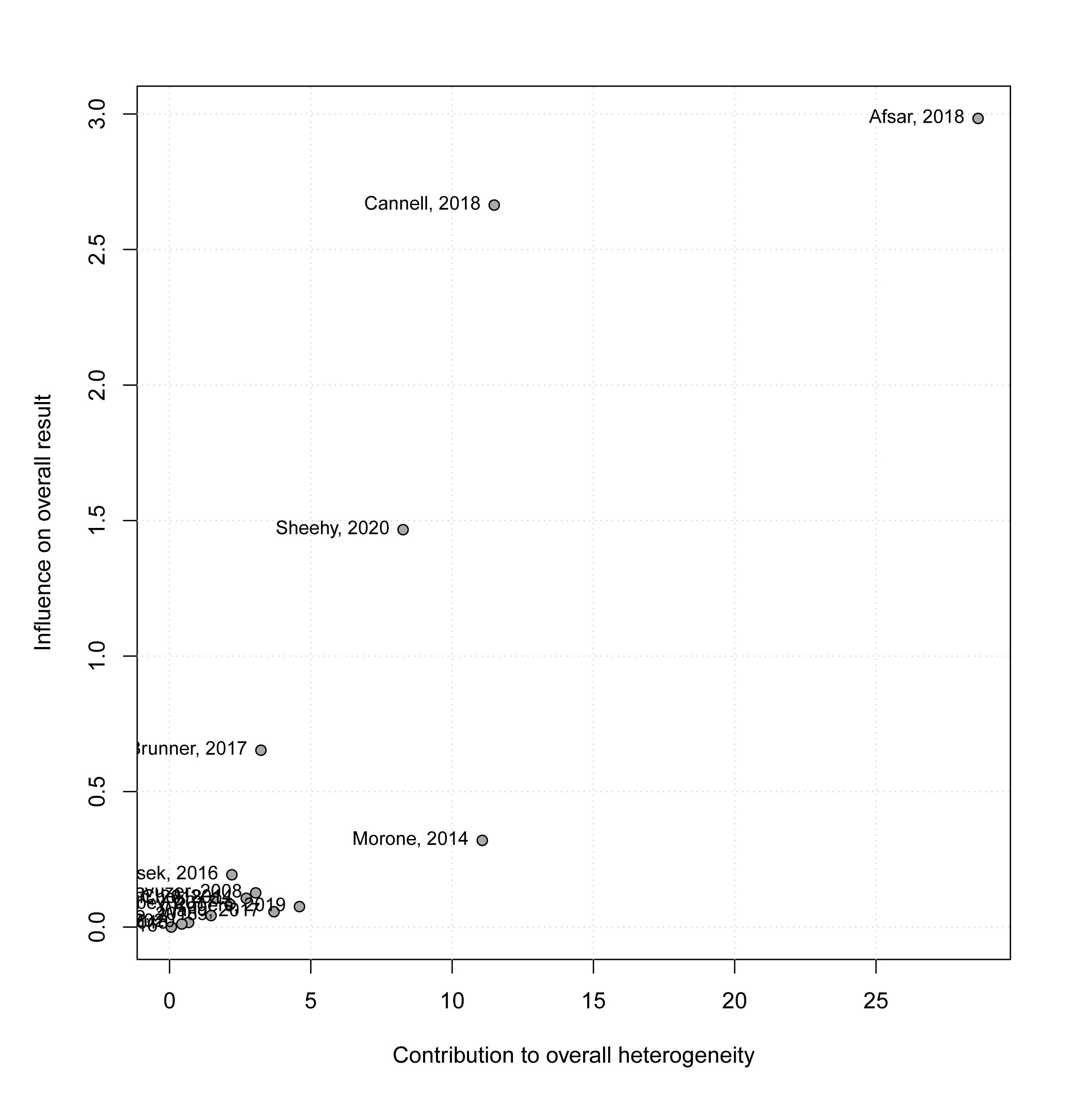

Supplement: Supplementary Figure 1 — Baujat plot showing the contribution of each study into the overall heterogeneity. [file Image_1.TIF]

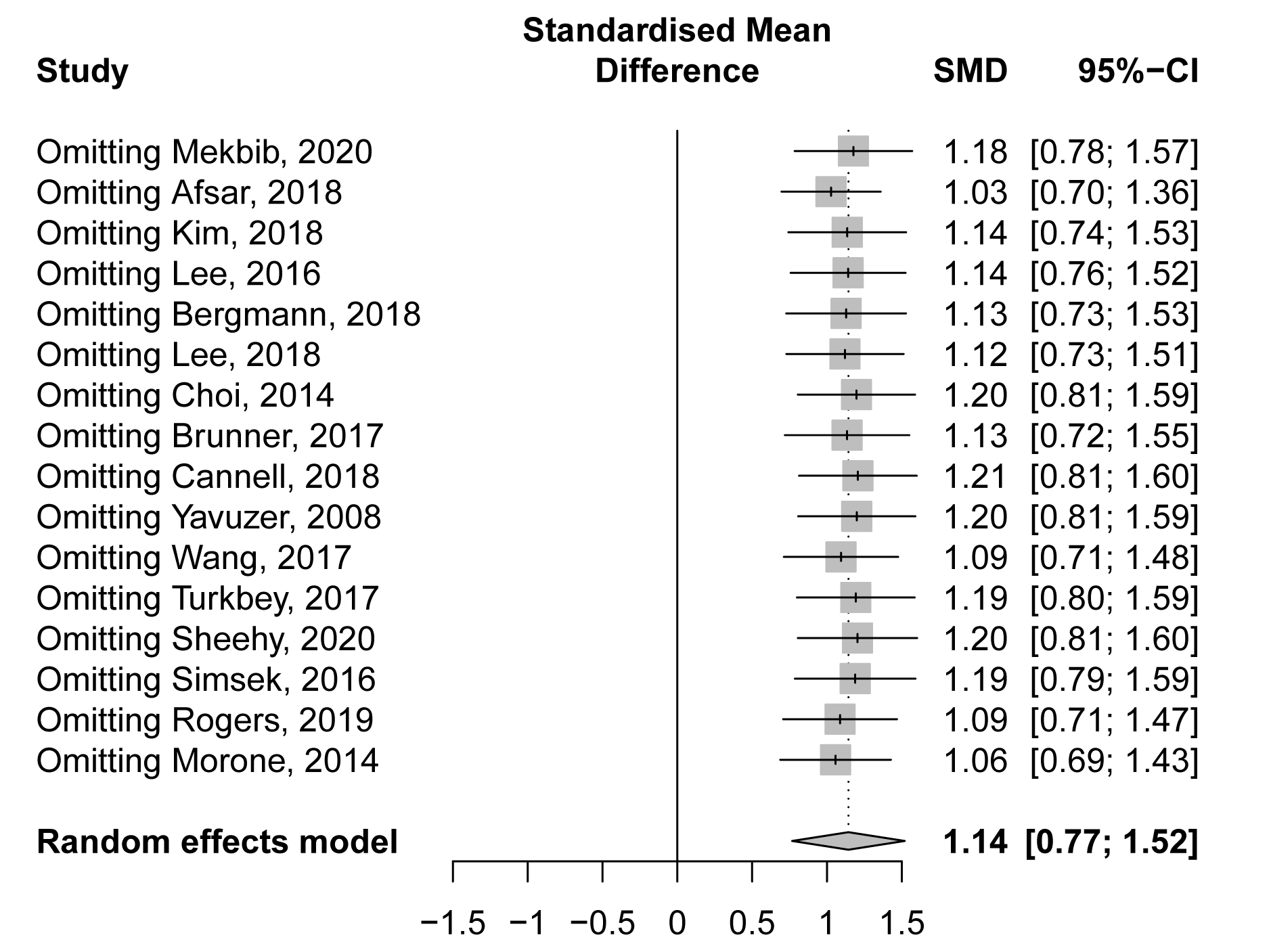

Supplement: Supplementary Figure 2 — Leave-one-out sensitivity analysis for the effectiveness of virtual reality–based rehabilitation (preintervention vs. postintervention). [file Image_2.TIF]

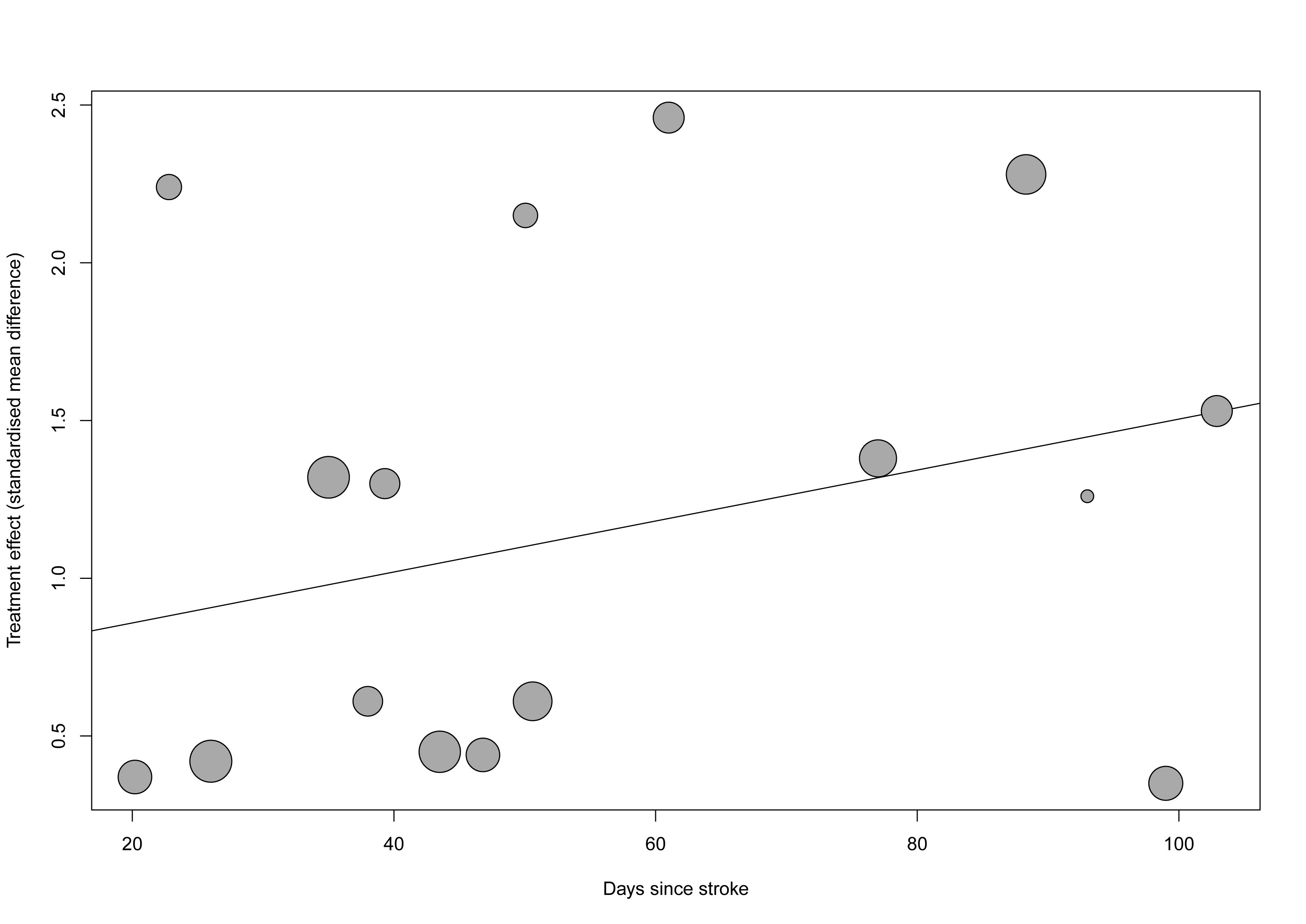

Supplement: Supplementary Figure 3 — Metaregression of the poststroke duration and virtual reality–based rehabilitation effectiveness (preintervention vs. postintervention). [file Image_3.TIF]

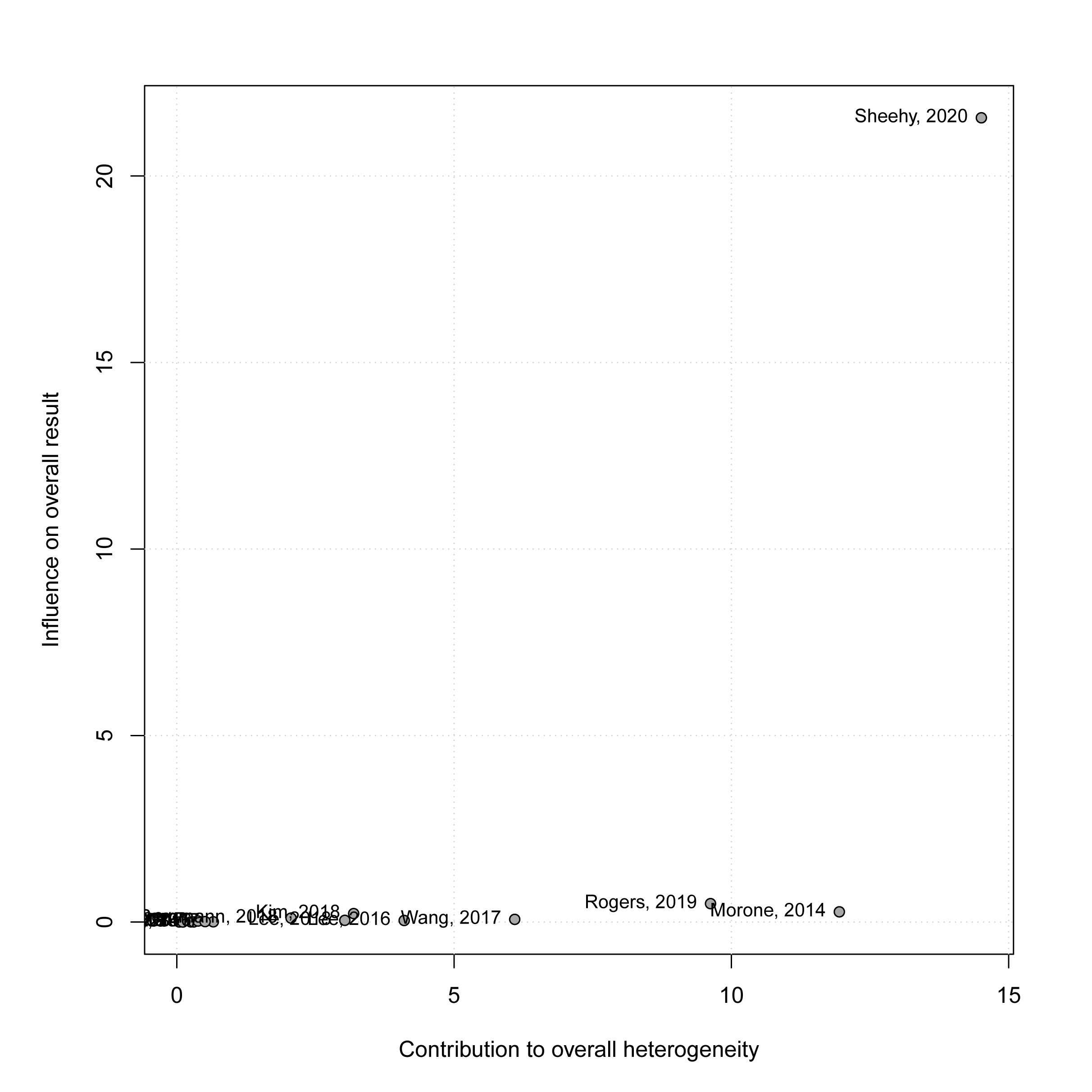

Supplement: Supplementary Figure 4 — Baujat plot showing the contribution of each study into the overall heterogeneity. [file Image_4.TIF]

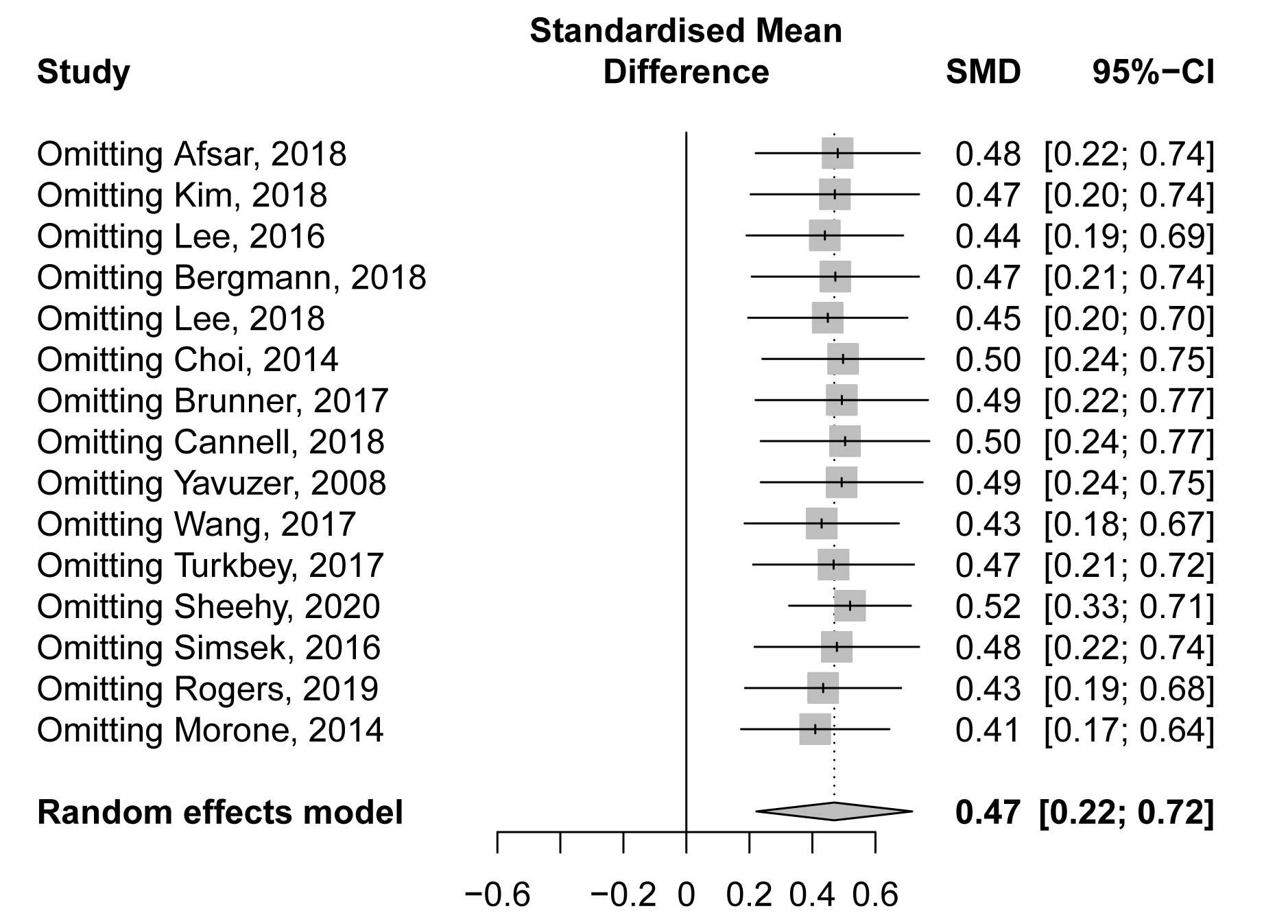

Supplement: Supplementary Figure 5 — Leave-one-out sensitivity analysis for the effectiveness of virtual reality–based rehabilitation (VR vs. conventional therapy). [file Image_5.TIF]

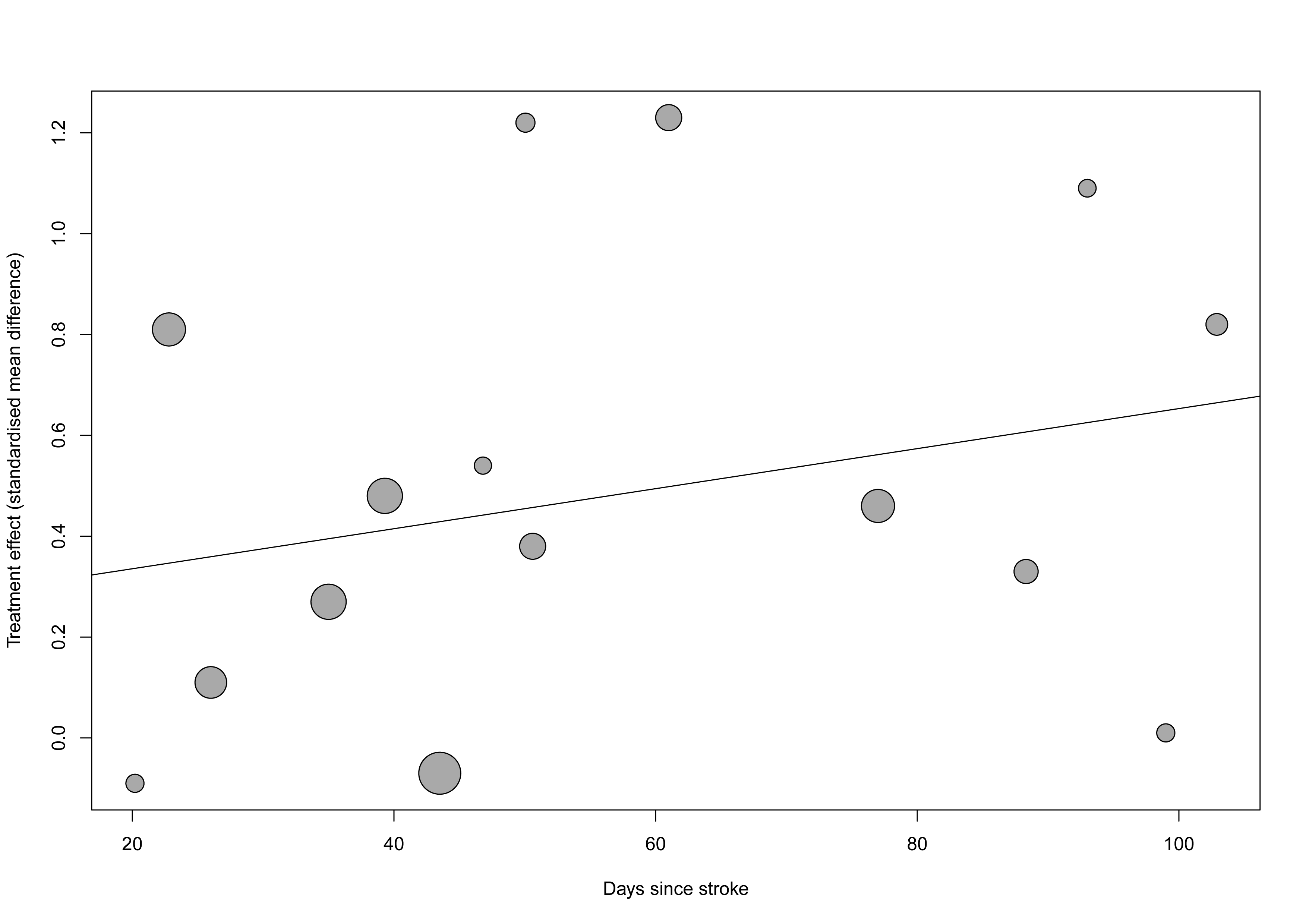

Supplement: Supplementary Figure 6 — Metaregression of the poststroke duration and virtual reality–based rehabilitation effectiveness (VR vs. conventional therapy). [file Image_6.TIF]
